# Eight-year scintigraphic follow-up of a patient with Chronic Recurrent Multifocal Osteomyelitis (CRMO): A case report

**DOI:** 10.22038/aojnmb.2025.89201.1646

**Published:** 2026

**Authors:** Mehrshad Abbasi

**Affiliations:** Department of Nuclear Medicine, Vali-Asr Hospital, Imam Hospital Complex, Tehran University of Medical Sciences, Tehran, Iran

**Keywords:** Chronic recurrent multifocal Osteomyelitis, Bone scintigraphy, Breast cancer

## Abstract

Chronic Recurrent Multifocal Osteomyelitis (CRMO) is a rare autoinflammatory bone disorder characterized by episodic bone pain, swelling, and radiologic evidence of sterile osteolytic lesions. We report the case of a 59-year-old woman (initial symptoms at age 51) with rheumatoid arthritis and remote triple-negative breast cancer who developed bilateral tibial lesions, observed serially over an eight-year follow-up period. The bone lesions appeared spontaneously, remained for variable durations, and regressed without specific treatment, paralleling pain severity. Laboratory data showed elevated inflammatory markers, while biopsy ruled out infection and malignancy. Bone scintigraphy and MRI confirmed multifocal active bone lesions, consistent with CRMO. The case highlights the diagnostic challenges of CRMO in adults, especially in the presence of other autoimmune conditions, and emphasizes the importance of long-term imaging follow-up. This case also illustrates that CRMO may mimic malignancy or infectious osteomyelitis, yet respond well to anti-inflammatory therapy alone. Early recognition can prevent unnecessary interventions.

## Introduction

 Chronic Recurrent Multifocal Osteomyelitis (CRMO) is an autoinflammatory bone disorder that predominantly affects children and adolescents (1, 2). However, it can occasionally present in adults, particularly in middle-aged women, often posing a diagnostic challenge due to its rarity in this age group (3). The disease is characterized by recurrent episodes of painful, sterile bone lesions that typically involve the metaphyseal regions of long bones (4). These lesions may resolve spontaneously or respond to anti-inflammatory therapies, only to reappear later at different skeletal sites-often on the contralateral limb (5).

 In adults, distinguishing CRMO from other conditions such as chronic infectious osteomyelitis, malignancy, or metastatic bone disease is particularly difficult, especially in individuals with underlying autoimmune or oncologic disorders (6). The diagnostic challenge is compounded in patients with a history of breast cancer, in whom new bone lesions raise immediate concern for metastatic spread.

 This report describes a rare case of an adult woman with a long-standing history of rheumatoid arthritis and triple-negative breast cancer who developed multifocal, migratory tibial bone lesions over an eight-year period. 

 The clinical and radiologic features were consistent with CRMO, and the patient experienced spontaneous remissions without specific treatment beyond maintenance immunosuppression for rheumatoid arthritis. This case highlights the importance of considering CRMO in the differential diagnosis of bone lesions in adults with complex medical histories.

## Case presentation

 A 59-year-old woman presented with excruciating pain localized to the left proximal tibia. She had a 20-year history of seropositive rheumatoid arthritis (RA), currently active, as evidenced by a rheumatoid factor (RF) of 95 IU/mL and C-reactive protein (CRP) of 6.5 mg/L. Her symptoms included disabling pain in the lower extremities, with minimal morning stiffness. At the time of presentation, she was being treated with weekly methotrexate injections (20 mg) and daily low-dose prednisolone (5 mg).

 The patient also had a significant oncologic history. Thirteen years prior, she had been diagnosed with triple-negative breast cancer of the left breast. She underwent total mastectomy, followed by completion surgery of the contralateral breast due to a familial pattern of disease. Histopathology confirmed the triple-negative phenotype. She subsequently received adjuvant chemotherapy and radiotherapy.

**Figure 1:**
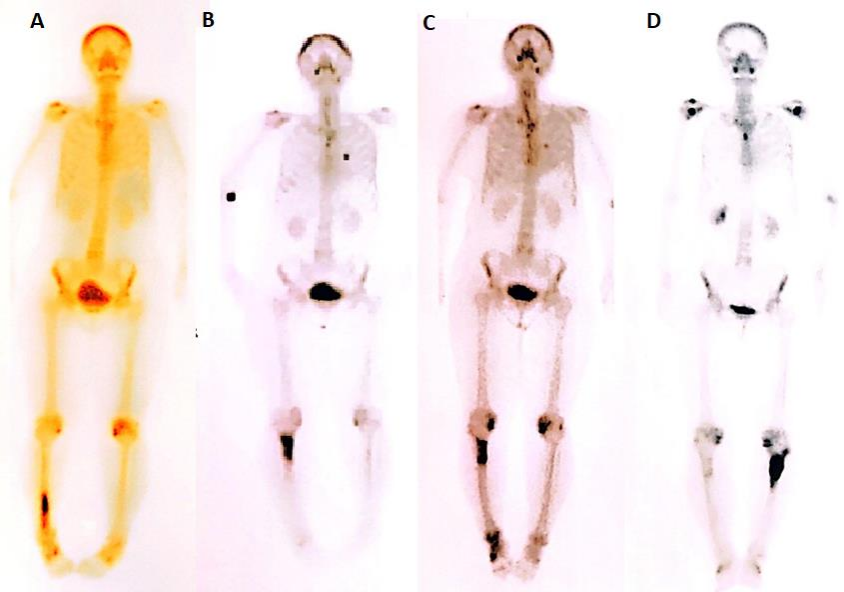
Eight-year scintigraphic timeline. (**A**) June 2018: focal uptake in the distal right tibial metaphysis/diaphysis during severe localized pain; biopsy revealed sterile inflammation. (**B**) January 2022: resolution of distal right tibial uptake with new intense activity in the proximal right tibial metaphysis corresponding to recurrent pain. (**C**) May 2023: persistent but decreased proximal right tibial uptake. (**D**) May 2025: new hyperactivity in the left proximal tibial metaphysis during severe pain; the prior right proximal site demonstrates a thin peripheral rim of uptake around a relatively hypoactive center, consistent with reparative change. Mild symmetric humeral head uptake was attributed to mechanical stress from crutch use. No anti-cancer therapy was administered during follow-up

 Approximately 5 years after completing cancer treatment, she began experiencing pain in the distal right tibia. A ^99m^Tc bone scan at the time (Figure 1; Panel A) showed a focal hot lesion, raising concern for bone metastasis. However, no targeted treatment was initiated due to a negative biopsy indicating sterile chronic inflammation. The pain in the distal tibia eventually resolved over the next two years.

 Subsequently, she developed pain in the proximal right tibia, again confirmed by bone scintigraphy to show focal hyperactive a large metaphyseal bone lesion (Panel B and C). Differential diagnoses included osteonecrosis and inflammatory bone disease. The lesion persisted for approximately three years, but eventually resolved without specific intervention.

 Seven years after the initial distal right tibial lesion, the patient developed severe pain in the left proximal tibia, centered at the metaphyseal-diaphyseal location. A repeat bone scan revealed a new hypermetabolic lesion in this location. Additionally, new uptake was noted in both humeral heads, which had not been present on prior scans. The humeral head findings were attributed to mechanical stress and possible subcapsular osteonecrosis, likely related to crutch use. Remarkable previous lesions in the right tibia had resolved completely in the distal part of tibia and remitted noticeably leaving a hypoactive metaphysical area with surrounding thin hyperactive rim in proximal tibia (Panel D).

 Given the recurrent, multifocal, and migratory nature of the lesions, along with the absence of infection or clear metastatic disease, the clinical picture was deemed consistent with Chronic Recurrent Multifocal Osteomyelitis (CRMO). 

 Notably, no abnormal finding was detected in the prior biopsy and no further biopsy was performed due to spontaneous resolution of previous lesions and consistent imaging evolution. The patient was subsequently treated with high-dose corticosteroids with partial improvement in pain.

## Discussion

 The presented case illustrates a diagnostically challenging scenario involving a rare condition-Chronic Recurrent Multifocal Osteomyelitis (CRMO)-in a patient with concurrent active rheumatoid arthritis (RA) and a history of triple-negative breast cancer (TNBC). The coexistence of inflammatory and oncologic diseases complicates the interpretation of new skeletal lesions, particularly when these are painful, focal, and demonstrate increased uptake on bone scintigraphy.

 A key step in the diagnostic process was the exclusion of metastatic disease, particularly given the patient’s history of TNBC. However, recurrence of triple-negative breast cancer after a prolonged remission period of over seven years is considered extremely rare, especially in the absence of systemic signs or imaging evidence of disease spread (7, 8). Furthermore, the spontaneous remission of two successive tibial lesions on the right side-first distal, then proximal-without oncologic treatment provides strong evidence against a malignant process retrospectively. It is highly unlikely that bone metastases would regress spontaneously and sequentially without specific intervention (9).

 Adult-onset CRMO also known as chronic nonbacterial osteomyelitis (CNO) increasingly recognized in rheumatology cohorts shows important differences from pediatric disease. In adults, lesions often cluster in the anterior chest wall (sternocostoclavicular region) and clavicle, with variable appendicular involvement, a relapsing–remitting course, and frequent coexisting immune-mediated disease (13, 14). 

 Our case-dominant metaphyseal-diaphyseal tibial involvement with migratory, self-limited flares over eight years in a middle-aged woman with seropositive RA-aligns with adult-onset CNO patterns reported in contemporary series, despite the absence of anterior chest wall disease. Recent adult CNO/CRMO cohorts and case series report heterogeneous imaging and treatment responses but consistently emphasize (i) sterile lesions, (ii) frequent autoimmune comorbidity, and (iii) good response to anti-inflammatories or corticosteroids, all of which were present in our patient (15, 16).

 CRMO is understood to be an auto-inflammatory bone disease, and increasing evidence supports an immunologic basis for its pathogenesis (4). In this context, the patient’s underlying rheumatoid arthritis may act as a facilitating or coexisting inflammatory condition, as CRMO has been observed more frequently in patients with other autoimmune diseases (2). Although CRMO is classically described in children and adolescents, adult-onset cases-especially in middle-aged women-have been reported, albeit rarely (10).

 Diagnosis of CRMO remains one of exclusion, particularly in adults. Infectious osteomyelitis and malignancy must first be ruled out through clinical evaluation, imaging, and where possible, histopathology demonstrating sterile inflammation (6). Biopsy remains the gold standard for confirming nonbacterial osteomyelitis, although in this case it was not pursued due to the characteristic pattern of spontaneous resolution and absence of radiologic progression. Regarding the imaging patterns that may help differentiate CRMO/CNO from malignancy, on ^99m^Tc-MDP bone scintigraphy CRMO/CNO typically demonstrates metaphyseal-diaphyseal hot foci that may be bilateral and migratory, sometimes with a peripheral rim or cortical-predominant uptake pattern reflecting periostitis, hyperostosis, and healing. These features contrast with the more random, often axial-predominant distribution of breast cancer metastases and their tendency to persist or progress over time (17). On MRI, CRMO commonly demonstrates bone-marrow edema, periostitis, and cortical thickening without a substantial soft-tissue mass; this favors an inflammatory process over metastasis, where cortical destruction and a measurable soft-tissue component are more typical. In our patient, the previously active right proximal tibial site later showed a thin hyperactive rim around a relative photopenic center on scintigraphy, concordant with reparative remodeling rather than a necrotic tumor core.

 Treatment of CRMO is based on disease severity. Nonsteroidal anti-inflammatory drugs (NSAIDs) are recommended first-line agents in mild to moderate cases. In more severe or refractory cases, systemic corticosteroids, tumor necrosis factor-alpha (TNF-α) inhibitors, and interleukin-1 (IL-1) antagonists have shown efficacy (11, 12). In this case, the flare in the left tibia was managed with high-dose prednisolone with symptomatic improvement.

 In terms of limitations, only the initial lesion underwent histopathological evaluation, which demonstrated sterile chronic inflammation; subsequent flares were diagnosed on clinical and imaging grounds. Although repeated biopsy is often discouraged in CRMO/CNO once infection and malignancy have been excluded, the absence of serial tissue confirmation remains a key limitation and prevents the diagnosis from being considered absolutely definitive. In addition, due to the retrospective nature of this report, serial inflammatory markers were incompletely recorded, limiting correlations between symptoms, imaging activity, and systemic inflammation. Finally, pain intensity and functional status were documented qualitatively in routine clinical notes rather than with standardized instruments (e.g., VAS or PROMIS), reducing the objectivity of treatment response assessment. Future prospective studies of adult CRMO should incorporate such standardized measures.

## Conclusion

 This case highlights a rare presentation of Chronic Recurrent Multifocal Osteomyelitis (CRMO) in an adult patient with complex comorbidities, including rheumatoid arthritis and a history of breast cancer. The recurrent and migratory nature of sterile bone lesions, along with spontaneous remission and response to anti-inflammatory therapy, support the diagnosis. Given its rarity in adults and its radiologic resemblance to metastatic or infectious bone disease, CRMO should be considered in the differential diagnosis of multifocal bone lesions-especially in patients with autoimmune backgrounds. Early recognition may prevent unnecessary interventions and allow effective treatment with immunomodulatory and anti-inflammatory agents.
